# Nondestructive Evaluation of Wet Aged Beef by Novel Electrical Indexes: A Preliminary Study

**DOI:** 10.3390/foods8080313

**Published:** 2019-08-02

**Authors:** Shinobu Ihara, Md Zohurul Islam, Yutaka Kitamura, Mito Kokawa, Yeun-Chung Lee, Suming Chen

**Affiliations:** 1 Graduate School of Life and Environmental Sciences, University of Tsukuba, 1-1-1, Tennodai, Tsukuba, Ibaraki 305-8572, Japan; 2 Department of Bio-industrial Mechatronics Engineering, National Taiwan University, No. 1, Sec. 4, Roosevelt Rd., Taipei 10617, Taiwan; 3 Faculty of Life and Environmental Sciences, University of Tsukuba, 1-1-1, Tennodai, Tsukuba, Ibaraki 305-8572, Japan

**Keywords:** electrical impedance spectroscopy (EIS), meat quality, texture, electrode, optical microscopy

## Abstract

The aim of this study was to investigate the suitability of electrical impedance spectroscopy (EIS) as a nondestructive quality monitoring tool of aged beef, focusing on the development of accurate electrical indexes. The relationship between the electrical indexes derived from the impedance ratio (IR) or admittance was established. Quality parameters such as the drip loss, cooking loss, water-holding capacity, and shear force of beef loin wet-aged for 0 to 21 days were evaluated to develop the new electrical indexes. In addition, the predictive capability of EIS was trialed using different indexes and frequencies. This study revealed that the most appropriate choice is to use electrical parameters at a lower frequency to determine or predict the physical properties of aged beef. The IR was derived from the ratio between the electrical impedance measured parallel to and perpendicular to the muscle fibers in the low-frequency domain. Furthermore, the degradation of muscle fibers was observed by optical microscopy. The investigated electrical indexes had higher correlations with shear force (0.52 ≤ *R*^2^ ≤ 0.58) compared to correlations with aging days (0.34 ≤ *R*^2^ ≤ 0.39). The findings of the study could be used for meat quality inspection in slaughterhouses as well as during aging.

## 1. Introduction

In the meat industry, consumers’ interest in meat quality parameters such as appearance, flavor, and nutrients is increasing [1]. Thus, meat suppliers must meet consumers expectations by ensuring product quality and supplying critically graded meat products at reasonable prices. However, traditional quality evaluation methods are time-consuming, complex, and destructive. For this reason, there is a strong demand to develop reliable online tools to monitor meat rapidly, nondestructively, and easily [2]. Electrical impedance spectroscopy (EIS) is an analytical method used to determine the electrical properties of meat by inducing an alternating electrical current [3]. It is known that the electrical properties of meat are dependent on frequency and strongly correlated with other physical properties such as the drip loss, color, texture, water-holding capacity, and share forces of the aged materials [1]. Therefore, EIS is widely used for the quality monitoring of various foods, including not only lean meat and processed meat products but also fruits and vegetables [1].

Meat tenderness is one of the main factors affecting the palatability of fresh meat [4]. In this regard, meat aging is a common process employed by the beef industry to ensure meat tenderness. Many studies have reported EIS as a nondestructive tool for the assessment of meat quality [2,4]. Although the detailed mechanism is still uncertain, it is well known that there are changes in extra- and intracellular electrolytes during the aging process due to the disruption of the cell membrane [5,6]. In particular, the changes in electrical properties are caused by a decrease in electrical impedance in the radiofrequency domain [1,2]. Due to the existence of anisotropy, electrical properties change with the direction of the electrical field during rigor mortis and early aging days [1,7]. However, during aging, the muscle fibers are broken down by enzymes and changes in pH and ion concentration occur [4,5,6]. This phenomenon causes the disappearance of anisotropy, decreasing the difference in electrical characteristics between different directions of the electrical field. Swatland [8] and Lepetit et al. [9] determined the impedance ratio (IR), which is defined as the ratio of impedance values perpendicular to and parallel to the muscle fibers when an alternating current is applied. According to the results of Lepetit et al. [9], meat aging decreased the IR in the radiofrequency domain, which showed good correlation with the tenderness of bovine meat. In other studies, meat quality in terms of texture was predicted by using the IR, as the dielectric nature and aging of muscle fibers contributes to the decrease in anisotropy [5,9]. On the other hand, Castro-Giraldez [10] developed Al^σ^, an electrical index derived from the conductance at radiofrequency, to determine the quality of pork. Their results indicated that Al^σ^ decreased with aging and showed a high correlation with the amount of free amino acids as well as the K value, an indicator of the amount of ATP-related compounds. This parameter could also be used to determine the quality of beef.

The aim of this study was to develop a more reliable electrical index for the quality monitoring of aging beef composed of all the electrical properties in the low-frequency domain. To determine the effectiveness of the new electrical indexes, they were compared with conventional indexes such as aging period and physical properties. Furthermore, the ultrastructure of the muscle fibers was observed by a microscope to unveil the mechanism of changes in electrical properties.

## 2. Materials and Methods

### 2.1. Materials and Aging Treatment

**Figure 1:**
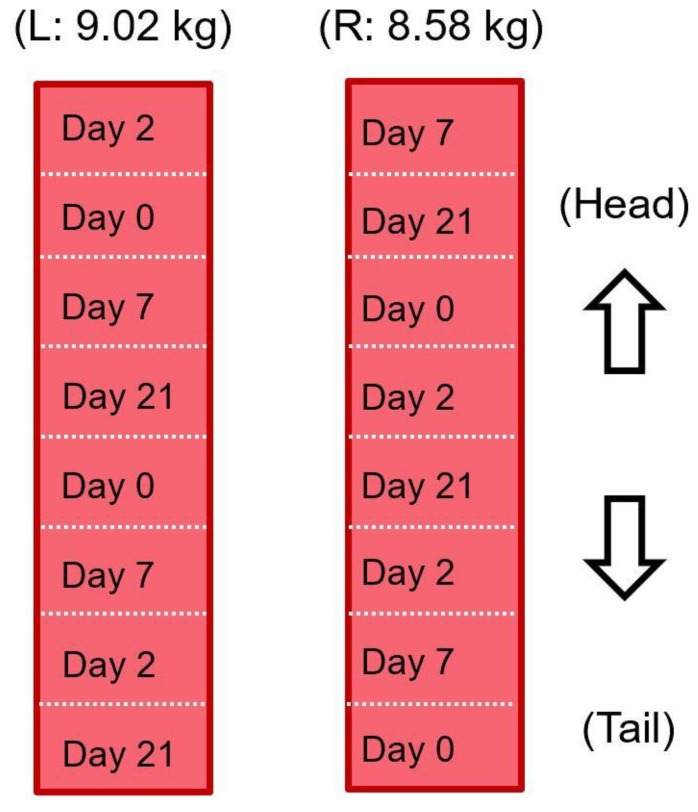
Sectioning of the materials. L and R indicate the left side and right side of the carcass, respectively.

Beef loins (longissimus dorsi of *Bos taurus* from Holstein; boneless) from both sides of one animal were used, at 2 days postmortem. They were obtained from the local meat processing plant at Ibaraki Prefecture, Japan. For aging, each loin was divided into eight parts of equal weight (about 1081.0 ± 147.0 g) and vacuum-sealed in a plastic bag for a total of 16 samples. Four samples balanced per side were randomly selected and assigned to a specific aging day (0, 2, 7, or 21 days) as presented in Figure 1. In this study, the aging period was counted from the day the samples were obtained (day 0 indicates 2 days postmortem). All samples were stored in a laboratory refrigerator controlled at 4.0 °C. Two 6 cm cubes were used to measure the cooking loss, texture (by Warner–Bratzler shear force), drip loss (as mentioned in Equation (13), and electrical properties. One single cube per aging day was used for histological analysis. The water-holding capacity was determined using 0.5 g of meat. All the subsequent analyses were done using fresh samples.

### 2.2. Measurement of Electrical Properties

**Figure 2:**
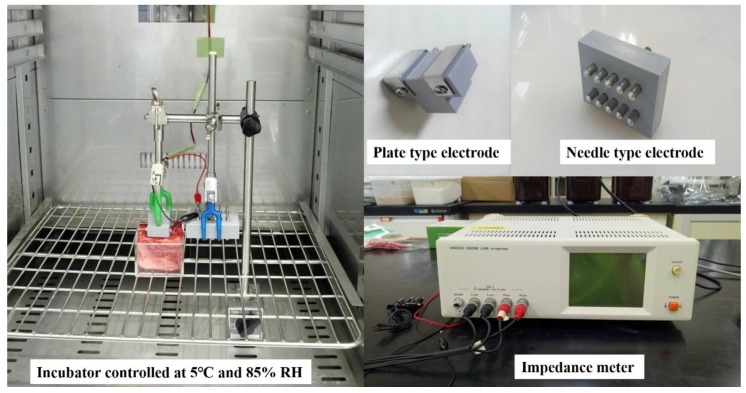
Measurement of electrical properties of aged beef.

To compare the effect of electrode shape, two types of electrodes—(A) plate and (B) needle—were used, as shown in Figure 2 [9]. For both electrodes, the parts in contact with the meat sample were made of stainless steel. The distances between the plates on the plate-type electrode and the rows of needles on the needle-type electrode were 10 mm and 20 mm, respectively. In order to avoid differences in measuring conditions that may affect the electrical properties (such as temperature differences), all the electrical properties were measured inside an incubator (EYELA KCL-2000, TOKYO RIKAKIKAI CO, LTD, Tokyo, Japan) fixed at 5 °C with 85% relative humidity [11]. The impedance meter, HIOKI 3532 HiTester (HIOKI E.E. CORPORATION, Nagano, Japan), two types of electrodes, and the sample were connected as shown in Figure 2. The 6 cm cube material was placed in a methacrylate cube to prevent changes in shape and the impedance of each sample was measured by using 1 V AC. Moreover, to determine the changes in electrical properties with respect to frequency, the impedance |Z| values were measured at 100 frequencies from 42 to 5 MHz, at logarithmic increments. Measurements were made at two different fiber directions, i.e., perpendicular to and parallel to the muscle fibers, to determine the transverse (TRANS) and longitudinal (LONG) impedances.

### 2.3. Electrical Indexes

TRANS and LONG |Z| values were measured at 100 frequencies between 42 Hz and 5 MHz at logarithmic increments, and the IR values (Equation (1)) were determined for each frequency. Next, the IR values in the low-frequency domain (42 to 10.79 kHz) were combined into two indexes, the integrated impedance ratio (IIR, Equation (2)) and the normalized impedance ratio (NIR, Equation (7). The relationships among these two electrical indexes and physical properties and aging days were analyzed. Furthermore, as a reference, changes in Mod Al^Y^ (Equation (9), which indicate the difference of admittance in the low- and high-frequency domains, were also analyzed.

#### 2.3.1. Impedance Ratio (IR)

IR is defined as the ratio between impedance values measured perpendicular to and parallel to the muscle fibers, as shown in Equation (1):

(1) $$IR=\left|Z_{TRANS}\right|/\left|Z_{LONG}\right|.$$

#### 2.3.2. Integrated Impedance Ratio (IIR)

When the IR is described as the function of frequency, the integrated impedance ratio (IIR) can be determined by integration with respect to the frequency in the range of 42 to 10.79 kHz. The relationship between IR and IIR is described by Equation (2):

(2) $$\mathrm{IIR}\;\left(Hz\right)=\overset{10.79\;kHz}{\underset{42\;Hz}{\int }}IR\;\;df.\;\;\;\;\;\;\;$$

The IR values of 48 points from 42 to 10.79 kHz were applied to determine the IIR by trapezoidal rule; hence, the area of each of the 47 trapezoids can be calculated numerically and combined by the equations below:

(3) $$\mathrm{IIR}\;\left(Hz\right)≒S_{1}+S_{2}+\cdots +S_{n}+\cdots +S_{47},$$

(4) $$S_{n}=\frac{\left(IR_{n}+IR_{n+1}\right)\times \left(f_{n+1}-f_{n}\right)}{2},$$

Here, *n* = 1, 2, … 47. Where IIR is the integrated impedance ratio (Hz); *S_n_* is the area of the *n*th trapezoid (Hz); *f_n_* is the frequency at the *n*th point (Hz). Here, the range from 42 to 5 MHz is equally and logarithmically divided into 100 points by Equation (5):

(5) $$F=\frac{\log_{10}\left(5\times 10^{-6}\;Hz\;-42\;Hz\right)}{100-1}.$$

The relationship between the integral number *n* (n=1, 2,…, 47) and the frequency is defined by Equation (6) in this analytical method:

(6) $$f_{n}=10^{\left(\left(n-1\right)F+\log_{10}42\;Hz\right)}.$$

#### 2.3.3. Normalized Impedance Ratio (NIR)

The mean IR in the low-frequency domain is defined as the normalized impedance ratio (NIR) and is calculated using the following equation:

(7) $$NIR=\overset{48}{\underset{i=1}{\sum }}IR_{i}=\frac{IR_{42\;Hz}+\ldots +IR_{10.79\;kHz}}{48},$$

where NIR is the normalized impedance ratio and IR is the impedance ratio.

The result of the IIR greatly depends on the frequency because the increment in the high-frequency domain is larger than that in the low-frequency domain. On the other hand, all IR values are normalized and do not depend on the frequency when the mean IR is calculated via the NIR method.

#### 2.3.4. Modified Al^Y^ (Mod Al^Y^)

Al^σ^, as investigated in a previous study [10,12] and shown in Equation (8), was modified and defined as Modified Al^Y^ (Mod Al^Y^), as shown in Equation (9). This index was used as a reference in comparison with the IIR and NIR by examining their correlations with aging days and physical properties. According to the previous study of Castro-Giraldez et al. [10,13], the quality of pork was determined by calculating the Al^σ^ from conductance values at 1 and 300 kHz (Equation (8).

(8) $$Al^{\sigma }=\left[\frac{\sigma_{300\;kHz}-\sigma_{1\;kHz}}{\sigma_{300\;kHz}}\right]\times 100,$$

where σ indicates the conductance (Ω^−1^). However, only slight differences in the conductance between 1 and 300 kHz were observed in the aging beef. Moreover, changes in the electrical properties of beef with changing frequency are composed of not only resistance but also capacitance and reactance. Capacitance is an especially important parameter due to the fact that it acts as an insulator when an alternating electrical current is applied [1,2]. Therefore, the admittance measured at 96 Hz and 3.12 MHz was used to highlight the frequency dependence in this study.

(9) $${\mathrm{Mod}\;\mathrm{Al}}^{Y}=\left[\frac{Y_{3.12\;MHz}-Y_{96\;Hz}}{Y_{3.12\;MHz}}\right]\times 100,$$

where Y indicates the admittance (Ω^−1^), which is the inverse of impedance. Moreover, σ denotes the conductance. Both of these parameters are defined in Equations (10–12).

(10) $$\left|Z\right|=\;\sqrt{R^{2}+{\left|X_{C}-X_{L}\right|}^{2}},$$

(11) $$\sigma \;=\;\frac{1}{R}\;,$$

(12) $$Y=\frac{1}{Z},$$

where |Z| is the impedance (Ω); R is the resistance (Ω); X_c_ is the inductive capacitance (Ω); X_L_ is the inductive reactance (Ω); *σ* is the conductance (Ω^−1^); Y is the admittance (Ω^−1^).

### 2.4. Determination of Meat Quality Parameters

#### 2.4.1. Determination of Drip Loss

In this study, the drip loss of each sample was determined according to the method described by Honikel [13] with slight modifications. The following formula was employed:

(13) $$Drip\;loss\;\left(\%\right)=\left(1-\frac{W_{m}}{W_{m+b}-W_{b}}\right)\times 100,$$

where *W_m_* is the weight of the material after wiping off the drip (g); *W_m+b_* is the total weight of the vacuum bag and the material (g); *W_b_* is the weight of the vacuum bag after washing and drying (g).

#### 2.4.2. Water-Holding Capacity (WHC)

The WHC measurement was performed following the method reported by Saito [14]. A small sample piece of 0.5 g was used to measure WHC. After weight measurement, the sample was wrapped in a membrane filter (ADVANTEC, Japan; pore size: 10 μm; diameter: 47 mm), inserted into a 50 mL tube with 45 g of glass beads, and centrifuged at 4000× *g* for 30 min at 4 °C. Finally, the weight of the sample was measured after centrifuging. Equation (14) was used to determine WHC:

(14) $$WHC\;\left(\%\right)=\left(\frac{W_{ac}}{W_{bc}}\right)\times 100,$$

where *W_ac_* is the weight of the sample after centrifugation (g) and *W_bc_* is the weight of the sample before centrifugation (g).

#### 2.4.3. Texture and Cooking Loss

The texture and cooking loss were determined following the methods reported by Saito [14] and Wheeler and Papadopoulos [12], respectively. The 6 cm meat cubes were cooked in an incubator (Nexus WBX-270, AS ONE Corporation, Osaka, Japan) until the core temperature reached 71 °C. During heating, the meat cubes were individually placed into plastic bags and a thermocouple was inserted to monitor the temperature by a temperature logger, HIOKI LR 8432 (HIOKI E.E. CORPORATION, Nagano, Japan). After heating, the material was cooled with running water for at least 30 min to obtain uniform muscle fibers and prevent drip flow. Six cylindrical cores (diameter of 1.27 cm) were cut out from the meat block with a cylindrical cutter. Each core was sheared perpendicular to the muscle fiber orientation with a Warner–Bratzler shear force blade connected to a texture measurement apparatus (EZ-SX, SHIMADZU CORPORATION, Kyoto, Japan) with a 100 N compression load cell operating at a crosshead speed of 250 mm/min.

Cooking loss was determined by measuring the weight of the material before and after heating the 6 cm cube allocated for measuring shear force. Thus, Equation (15) was applied to determine the cooking loss:

(15) $$Cooking\;loss\;\left(\%\right)=\left(1-\frac{W_{ah}}{W_{bh}}\right)\times 100$$

where *W_ah_* is the weight of the sample after heating (g) and *W_bh_* is the weight of the sample before heating (g).

### 2.5. Histological Analysis

The muscle fibers were observed with an optical microscope following the method of Fukaya and Hukunaka [15]. As a pretreatment, the 1 cm cube materials were put into 30 mL of Bouin solution for at least 6 h to fix the sample, after which they were dehydrated by soaking in 30 mL of 70% ethanol solution for approximately 1 h. The fixed and dehydrated material was sliced using a microtome and dyed. Pictures were taken using an optical microscope, LEICA DM2500 (LEICA CAMERA AG, Wetzler, Germany), at 400× magnification to obtain typical examples of muscle fibers on each aging day.

### 2.6. Statistical Analyses

All experiments were carried out in at least triplicates and reported as the mean ± standard deviation of the independent measurements. Comparison of the means among different aging periods was determined by post hoc Tukey test. The significant level was set at *p* ≤ 0.05. One-way ANOVA was performed to evaluate the physical properties of the aged meat. The correlation coefficient (*R*^2^), coefficient of variation (CV), and *p*-value were determined by Origin Pro 8.5. software (Origin Lab Corporation, Northampton, MA, USA). The correlation presented in this study was calculated from the residual after adjustment of the fixed effects described in the ANOVA. Other statistical analyses were performed using Excel 2016 (Microsoft Corporation, Washington, DC, USA).

## 3. Results and Discussion

### 3.1. Drip Loss, Cooking Loss, WHC, and Shear Force

The effects of wet aging treatment on the physical properties of the meat (drip loss, WHC, and shear force) are shown in Table 1. The physical properties were significantly modified by aging. The WHC of beef significantly increased after 2 days of aging. Our WHC results followed the findings of Warner [16], where the WHC of meat initially decreased during the rigor period but increased with prolonged aging. During the postmortem period, the water located inside the muscle cells flows out to the extracellular water compartment due to the shrinkage of cytoskeletons and myofibrils. With prolonged aging, the water flows back inside of the muscle cells because of the defilement of the cytoskeletons and myofibrils [16]. This increase of water in the muscle cells may lead to an increase in WHC.

The drip loss was higher after 21 days of aging compared with 2 and 7 days, while day 0 showed intermediate values. However, the amount of drip loss was found very low compared with the values (1.88 ± 0.98%) reported by Byrne [4]. This may be due to the good packing system, in which the plastic bags holding the meat were shrunk after vacuum packing at the meat processing plant.

The cooking loss increased with the aging period, but the change was not significant (*p* > 0.05). Our results followed the findings of Shin et al. [17] and Lindahl et al. [18], who determined the cooking loss of meat and showed that it did not vary significantly during aging.

The shear force of the meat decreased dramatically with the prolonged aging period. The results indicated that the meat shear force decreased by approximately 50% after 7 days of aging. Similar results were reported by Lindahl et al. [18].

### 3.2. Electrical Parameters

#### 3.2.1. Impedance |Z| Value

**Figure 3:**
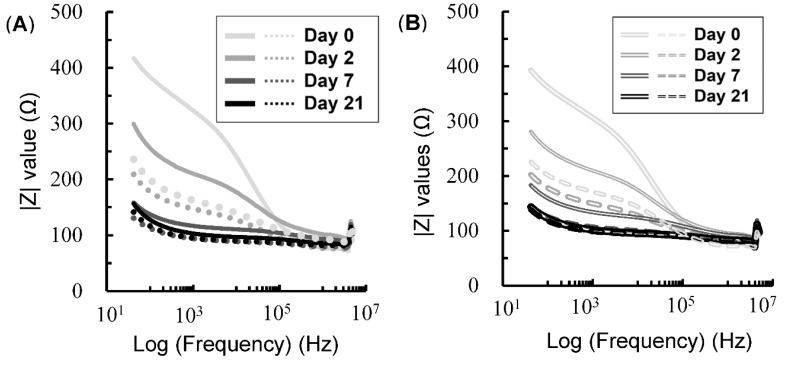
Changes in the mean |Z| values for the plate-type electrode (**A**) and needle-type electrode (**B**) over the aging period and over the frequency range from 42 to 5 MHz in longissimus dorsi. Solid and dot lines indicate |Z| values on transverse (TRANS) and longitudinal (LONG) impedances, respectively.

The changes in |Z| values with respect to aging days and frequency for the plate- and needle-type electrodes are presented in Figure 3A,B, respectively. In the low-frequency domain, the |Z_TRANS_| values were higher than the |Z_LONG_| values for all aging days due to the anisotropic structure [7,19]. Moreover, |Z| values decreased with aging regardless of the electrode type and the direction of electrical fields, especially in the low-frequency domain. This is because, at low frequencies, the current cannot pass through intact cells membranes, which act as capacitors. As aging proceeds, the disruption of cell membranes decreases their resistance, lowering the impedance of the electrical current. At relatively high frequencies, electric current flows through the extracellular fluid, membrane, and intracellular fluid [1]. This may be the reason for the smaller change in |Z| values over the aging period at frequencies higher than 10^5^ Hz. In summary, |Z| values decreased with prolonged aging period due to the degradation of the cell membranes as well as the increase in frequency, which leads to the transmission of electric current through the cell membrane.

#### 3.2.2. Impedance Ratio (IR)

**Figure 4:**
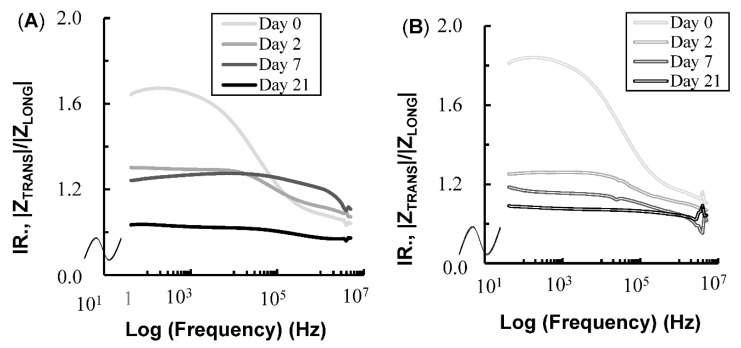
Changes in the mean IR values for the plate-type electrode (**A**) and needle-type electrode (**B**) over the aging period and over the frequency range from 42 to 5 MHz in longissimus dorsi.

The changes in the average IR values across aging days and frequencies for the plate- and needle-type electrodes are shown in Figure 4A,B, respectively. During early aging days and in the low-frequency domain, the IR values were large compared to those after prolonged aging and in the high-frequency domain. Large IR values indicate large differences between the impedances in the TRANS electrical field and those in the LONG electrical field due to anisotropy. The IR measured with both electrodes decreased dramatically between days 0 and 2, but the decrease was smaller after day 2. The decrease in IR indicates the disappearance of anisotropy [9,19].

Moreover, when the coefficient of variance (CV) for |Z| values and IR were compared, the CV of IR values were much lower than those of |Z| values. The average CV for IR and |Z| values were 0.141 and 0.294, respectively (not shown in the table). Because IR is calculated as the ratio between measurements made from different directions, variations in absolute impedance values can be cancelled, enabling the acquisition of more consistent data [9].

#### 3.2.3. Electrical Indexes

The changes in the three different types of electrical indexes with respect to aging days are presented in Table 2. The results demonstrated that the IIR, NIR, and Mod Al^Y^ of the meat decreased with increased aging. Significant differences were found between days 0 and 2 in IIR and NIR measured with the needle-type electrode, while changes in these two indexes were moderate and not significant in later aging days. On the other hand, there was no significant change in the IIR or NIR index measured by the plate-type electrode. No significant change was observed in the Mod Al^Y^ index for both plate- and needle-type electrode measurements throughout the aging period. These results show that the type of electrode and analysis method affect the correlation between aging days and electrical parameters. Moreover, the results of texture measurement showed that physical changes during the aging of beef mostly occurred during early aging days, and the changes become smaller as aging progresses. These results are in accordance with those of previous studies [4,7,19].

### 3.3. Correlations between Electrical Indexes, Physical Properties, and Aging Days

The correlation coefficients between the electrical indexes and physical parameters are presented in Table 3. There was a strong relationship between the electrical indexes and shear force (*R*^2^ values shown in Table 4), which agrees with the results of previous studies conducted by Byrne et al. [4], Damez et al. [7], and Lee et al. [16], who studied the electrical properties of musculus longissimus dorsi in beef. Damez et al. [7] and Quali et al. [6] reported that proteolysis caused by the aging treatment contributed to an increase in ion concentration, which led to enhanced conductivity (lower impedance) and increased tenderness of the meat.

**Figure 5:**
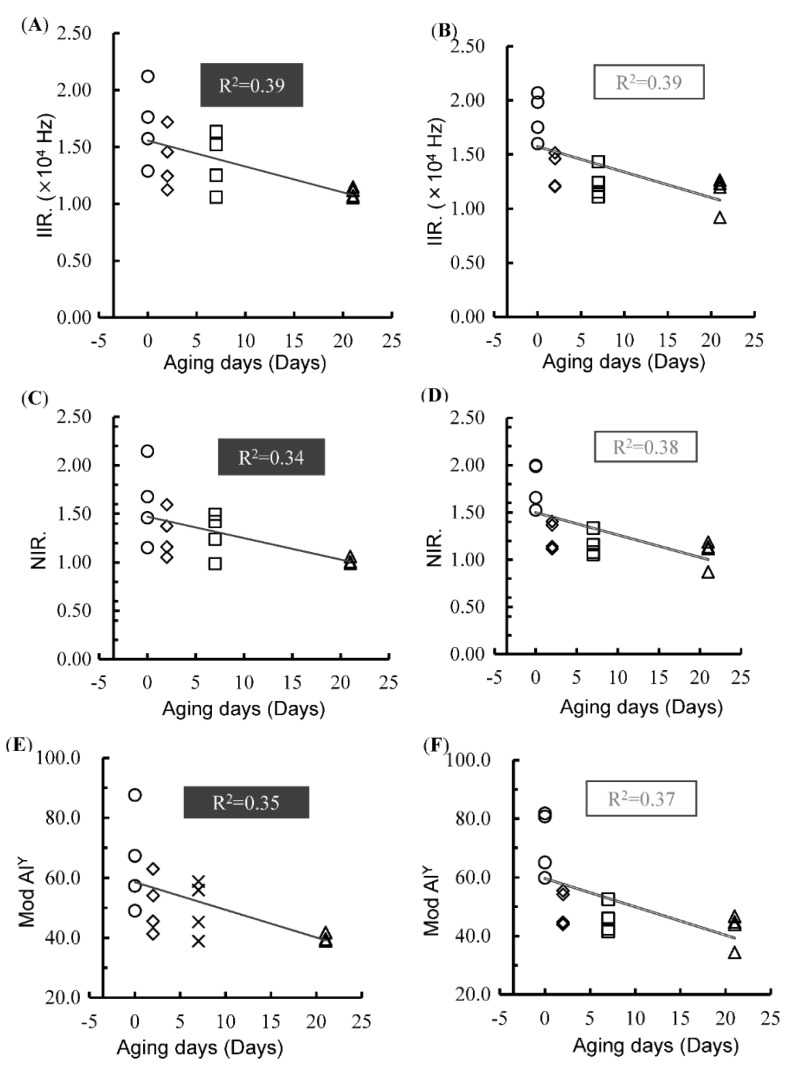
Variation of IIR measured by plate-type (**A**) and needle-type (**B**) electrodes; NIR measured by plate-type (**C**) and needle-type (**D**) electrodes; Mod Al^Y^ measured by plate-type (**E**) and needle-type (**F**) electrodes with aging days. Each different marker symbol (●, ◆, ■, ▲) indicates a specific aging day (0, 2, 7, 21), respectively.

It is interesting to note that the correlations between electrical indexes and shear force were higher than those between the same electrical indexes and aging days. Figure 5 shows the changes in electrical indexes with aging days. The results indicate that proteolysis during aging may not occur uniformly in longissimus dorsi. The results of the texture measurement also showed that physical changes in the aging meat within the same animal and the same muscle were dependent on the measuring position. A previous study also mentioned that there were significant differences in the shear force of aging beef within the same muscle [17]. Therefore, this phenomenon may contribute to changes and differences in the electrical indexes.

As shown in Table 3, there were low correlations between the electrical indexes and drip loss. The findings of the present study differed from the results reported by Lee et al. [16], who found that the electrical conductivity decreased with the increase of drip loss in pork loins. However, the previous study did not use vacuum packaging treatment for the materials, and it is possible that drip loss was affected by the packaging system and aging procedure.

Mod Al^Y^, as the conventional electrical index, showed a high correlation with the shear force, while IIR and NIR showed high correlations not only with shear force but also with aging days. Therefore, these two electrical indexes could be applied to determine the quality of aging beef.

### 3.4. Histological Analysis

**Figure 6:**
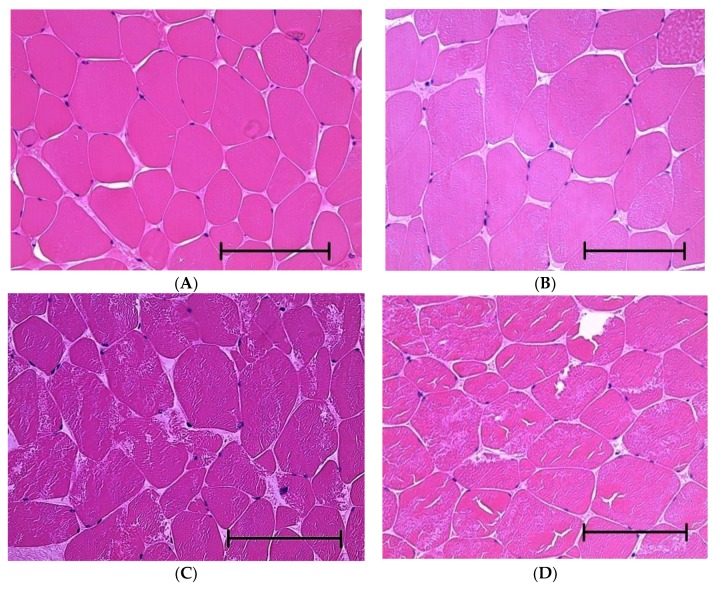
Typical images of muscle fibers of longissimus dorsi observed by optical microscope (×400). (**A**–**D**) indicate materials obtained on days 0, 2, 7, and 21, respectively. The scale bar indicates 100 μm.

The ultrastructural changes in the muscle fibers of longissimus dorsi during the aging period were investigated by optical microscopy. The images of muscle fibers acquired on days 0, 2, 7, and 21 are denoted A, B, C, and D, as shown in Figure 6. The myofibril and sarcoplasm were signified by the red and white dyed parts, respectively. Images of the muscle fibers on days 0 and 2 showed intact myofilaments composed of actin and myosin, indicated by cross-sections dyed completely red. On later aging days, the insides of the myofibrils were mottled and divided into red and white parts, suggesting that degradation occurred. Moreover, the change in the shape of myofibrils with aging days was obvious, because some of the myofibrils had disappeared with the progress of aging. During aging, connective proteins break down, causing structural changes such as the fragmentation of myofibrils and the degradation of cytoskeletons [1]. The results of the present study are also in agreement with a previous study reported by Ho et al. [17], who revealed that the denaturation of muscle fibers in beef during aging was caused by the effect of several enzymes, as determined by measuring I-band breaks. Taylor and Koohmaraie [18] also reported that I-band breaks and myofibril detachment from sarcolemma occurred during the aging of lamb meat.

**Figure 7:**
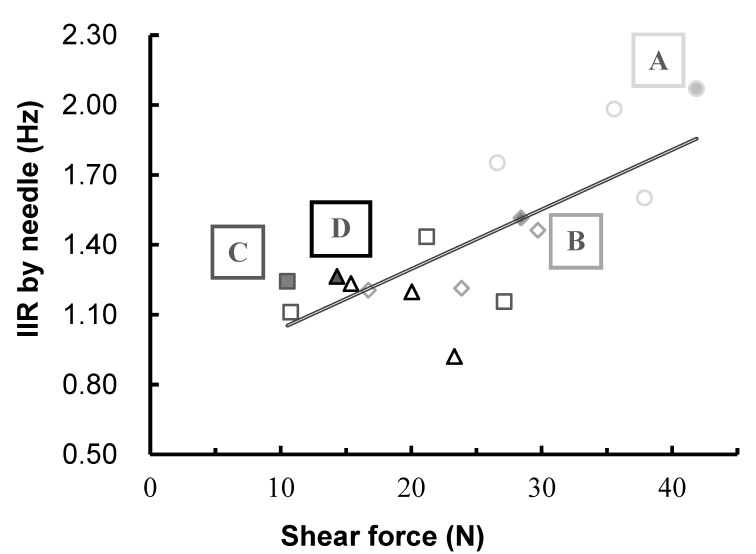
Variation of IIR by needle-type electrode with shear force during aging. Each different marker symbol (○, ◇, □, △) indicates a specific aging day (0, 2, 7, 21), respectively. The images shown in Figure 6 were acquired from the samples depicted here as filled markers labeled A, B, C, and D.

The relationship between shear force and the IIR electrical index during aging is shown in Figure 7. It also indicates the sample from which the images in Figure 6 were acquired. The data show that there is a relationship between the degradation of muscle fibers, the decrease in shear force values after 7 days, and the changes in the electrical properties of the meat. In previous studies, the degradation of muscle fibers and a simultaneous decrease in shear force was observed [20]. It has been reported that the activation of endogenous enzymes leads to an increase in the concentration of Ca^2+^ [21,22,23,24], and this further leads to changes in osmotic pressure, which alters the cell membranes during the aging period [16]. Furthermore, the collapse of the muscle cell membranes could contribute to the increase in the intra- and extracellular ion concentration. According to the findings of the present study and previous works, we hypothesized that the enzymatic degradation of muscle fibers during aging leads to an increase in ion concentration, which contributes to the decrease in shear force and electrical indexes. The degradation of muscle fibers may affect the electrical indexes directly, but the relationship between muscle degradation and changes in electrical indexes is still unclear. Since it is difficult to quantify muscle degradation using images taken by a microscope, SDS-PAGE and Western blotting [24] should be used in further work to unveil the effects of muscle fiber degradation on the changes of shear force and electrical indexes.

## 4. Conclusions

The use of electrical impedance spectroscopy (EIS) was investigated to determine the quality of beef during aging between 2 and 23 days postmortem. In the present study, two types of electrodes—plate- and needle-type electrodes—were used to measure the electrical impedance in two directions—parallel and perpendicular to the direction of meat fibers. The impedance ratio (IR) is defined as the ratio between impedance values measured perpendicular to and parallel to the muscle fibers, and the IR values measured at 100 different frequencies were combined into two parameters—the integrated impedance ratio (IIR) and the normalized impedance ratio (NIR). Furthermore, modified admittance (Mod Al^Y^) is defined as the difference between the admittance at relatively high and low frequencies. The correlation between quality parameters of beef and the three indexes—IIR, NIR, and Mod Al^Y^—were examined. In addition, muscle fibers were observed by optical microscopy to understand the effect of muscle fiber degradation on the changes in the electrical parameters and physical properties.

The results showed that changes in the electrical characteristics during the aging process of beef occur during early postmortem days and in the low-frequency domain. There was no definite superiority between the plate- and needle-type electrodes. The electrical indexes of IIR and NIR showed correlations with shear force, cooking loss, and water holding capacity (WHC), and they were also affected by aging days. However, measurements at different positions within the same muscle showed that aging treatment did not contribute uniformly to changes in physical properties. Although observations by optical microscopy inferred that muscle fiber degradation may contribute to changes in electrical and textual properties, quantitative analysis of the relationship between these properties was not possible. Thus, the following should be part of further work:

(1) Measurement of the changes in the electrical indexes of aging beef by using several types of electrodes and many samples in order to determine a more reliable nondestructive measuring method.
(2) Quantification of the degradation of muscle fiber during aging by using methods such as SDS-PAGE and Western blotting. Analysis with these methods may help to elucidate the mechanism of changes in drip loss and WHC during the aging period as well as to clarify the relationship between physical properties and electrical indexes.

## Figures and Tables

**Table 1:** Effect of the number of wet aging days on drip loss, cooking loss, water-holding capacity (WHC), and shear force in longissimus dorsi. The values are mean ± standard deviation (SD) of three independent measurements.

Parameter	Aging Period (Days)				*p*-Value
	0	2	7	21	
Drip loss (%)	0.6 ± 0.16 ^a,b^	0.3 ± 0.10 ^a^	0.3 ± 0.10 ^a^	0.9 ± 0.10 ^b^	<0.01
Cooking loss (%)	18.8 ± 1.85	19.5 ± 2.01	19.8 ± 0.88	21.0 ± 0.80	>0.05
WHC (%)	71.8 ± 2.67 ^b^	83.0 ± 3.93 ^a^	78.4 ± 2.48 ^a^	80.0 ± 3.20 ^a^	<0.05
Shear force (N)	35.5 ± 6.45 ^a^	24.7 ± 5.87 ^a,b^	17.4 ± 8.17 ^b^	18.3 ± 4.19 ^b^	<0.01

The means with different lowercase superscripts in a row differ significantly by Tukey test; α = 0.05.

**Table 2:** Changes in the Integrated Impedance Ratio (IIR), Normalized Impedance Ratio (NIR), and Modified (Mod) Al^Y^ with aging days by plate- and needle-type electrodes in longissimus dorsi.

Electrical Index		Aging Period (Days)			
		0	2	7	21
IIR (×10^4^ Hz)	Plate *	1.69 ± 0.35	1.39 ± 0.26	1.37 ± 0.26	1.10 ± 0.04
	Needle *	1.85 ± 0.21 ^a^	1.35 ± 0.16 ^b^	1.24 ± 0.14 ^b^	1.15 ± 0.16 ^b^
NIR	Plate *	1.63 ± 0.39	1.30 ± 0.24	1.26 ± 0.24	1.03 ± 0.04
	Needle *	1.79 ± 0.24 ^a^	1.26 ± 0.15 ^b^	1.16 ± 0.13 ^b^	1.08 ± 0.14 ^b^
Mod Al^Y^	Plate	68.7 ± 25.05	47.8 ± 25.17	35.7 ± 23.61	33.6 ± 7.79
	Needle	65.1 ± 22.91	50.7 ± 23.26	45.0 ± 12.51	31.4 ± 9.01

* Indicates *p* < 0.05 by ANOVA. Different letters indicate that results were different significantly within the same row by Tukey test; α = 0.05 ± SD.

**Table 3:** Correlation coefficients (*R* values) between electrical parameters and physical parameters.

Electrical Index		Aging Days	Shear Force ^A^	Drip Loss	Cooking Loss	WHC
IIR	Plate	−0.616 *	0.760 **	−0.424	−0.567 *	−0.527 *
	Needle	−0.626 *	0.751 **	−0.061	−0.478	−0.636 *
NIR	Plate	−0.585 *	0.722 *	−0.401	−0.594 *	−0.530 *
	Needle	−0.616 *	0.754 **	−0.051	−0.509 *	−0.660 *
Mod Al^Y^	Plate	−0.591 *	0.748 **	−0.355	−0.599 *	−0.565 *
	Needle	−0.605 *	0.750 **	−0.050	−0.529 *	−0.667 *

^A^: the mean value of six measurements from each material was regarded as one result. * and ** indicate *p* < 0.05 and *p* < 0.01, respectively.

**Table 4:** Coefficient of determination (*R*^2^ value) between IIR, NIR, and Mod Al^Y^ measured by plate-type and needle-type electrodes with shear force over the aging period from 0 to 21 days.

Electrical Index	Type of Electrode	*R*^2^ Value
IIR (×10^4^ Hz)	Plate	0.58
	Needle	0.56
NIR	Plate	0.52
	Needle	0.52
Mod Al^Y^	Plate	0.56
	Needle	0.56
